# STAT3 for Cardiac Regenerative Medicine: Involvement in Stem Cell Biology, Pathophysiology, and Bioengineering

**DOI:** 10.3390/ijms21061937

**Published:** 2020-03-12

**Authors:** Shu Nakao, Tasuku Tsukamoto, Tomoe Ueyama, Teruhisa Kawamura

**Affiliations:** 1 Department of Biomedical Sciences, College of Life Sciences, Ritsumeikan University, Kusatsu 525-8577, Japan; snakao@fc.ritsumei.ac.jp (S.N.); gr0279ie@ed.ritsumei.ac.jp (T.T.); ueyama-t@fc.ritsumei.ac.jp (T.U.); 2 Ritsumeikan Global Innovation Research Institute, Ritsumeikan University, Kusatsu 525-8577, Japan

**Keywords:** JAK/STAT signaling, pluripotent stem cells, differentiation, cardiomyocytes, regenerative medicine

## Abstract

Heart disease is the most common cause of death in developed countries, but the medical treatments for heart failure remain limited. In this context, the development of cardiac regeneration therapy for severe heart failure is important. Owing to their unique characteristics, including multiple differentiation and infinitive self-renewal, pluripotent stem cells can be considered as a novel source for regenerative medicine. Janus kinase/signal transducer and activator of transcription 3 (JAK/STAT3) signaling plays critical roles in the induction, maintenance, and differentiation of pluripotent stem cells. In the heart, JAK/STAT3 signaling has diverse cellular functions, including myocardial differentiation, cell cycle re-entry of matured myocyte after injury, and anti-apoptosis in pathological conditions. Therefore, regulating STAT3 activity has great potential as a strategy of cardiac regeneration therapy. In this review, we summarize the current understanding of STAT3, focusing on stem cell biology and pathophysiology, as they contribute to cardiac regeneration therapy. We also introduce a recently reported therapeutic strategy for myocardial regeneration that uses engineered artificial receptors that trigger endogenous STAT3 signal activation.

## 1. Pluripotent Stem Cells and Their Characteristics

Pluripotent stem cells (PSC) are a cell type characterized by unlimited self-renewal and pluripotency. Owing to these cellular properties, PSCs, including embryonic stem cells (ESCs), epiblast stem cells (EpiSCs), and induced pluripotent stem cells (iPSCs), have been extensively studied for advancing regenerative medicine, including cell therapy with or without gene engineering. Over the past decade, numerous efforts have been made to address PSC characteristics that vary based on spatiotemporal regulation in early embryos. During embryonic development, the cells derived from the inner cell mass in the blastocyst—the origin of ESCs—remain partially pluripotent until the post-implantation epiblast stage, but, then, gradually differentiate toward later developmental stages [1]. There are two major states of pluripotency observed in mouse ESCs (mESCs) and EpiSCs (mEpiSCs): the former is isolated from the pre-implantation embryo and is termed the naïve PSC; the latter is derived from the post-implantation epiblasts and termed the primed PSC. The common characteristics of pluripotency are the ability to differentiate into the three germ layers, indicated by marker gene expressions in vitro, and to form teratomas in vivo [2,3,4]. In addition, naïve PSCs are germline competent and can form germline-transmitting chimeric mice, whereas primed PSCs often fail to produce chimeras. In contrast to dome-shaped naïve mESCs, human ESCs (hESCs) are typically similar to primed mEpiSCs, exhibiting a flat shape in colony morphology. Various omics technologies have been developed in the past ten years, and these have successfully identified a molecular signature for each type of pluripotency. The gene expression profiling of PSCs also revealed a dozen transcription factors and surface protein characteristics of the naïve and primed states [5,6]. The naïve pluripotency of the inner cell mass in the blastocyst presents only for a limited period during development. Determining the gene expression profiles during embryonic development and how they are dynamically changed afterward is promising for the identification of the novel molecules or signal pathways involved in the pluripotency signature, other than Oct4, Sox2, and Nanog, which are common markers of PSCs. One critical pathway is the Janus kinase/signal transducer and activator of transcription 3 (JAK/STAT3) pathway, which has been utilized for the acquisition and maintenance of pluripotency in culture.

In 2006 and 2007, Takahashi and Yamanaka first reported that ESC-like pluripotency could be induced by the ectopic expression of the master transcription factors OCT4, SOX2, KLF4, and c-MYC, resulting in iPSC production from murine and human somatic cells [7,8]. Although the use of hESCs has faced ethical and legal hurdles involving the use of human embryos, increasing the availability of human iPSCs (hiPSCs) will overcome these issues. The scientific breakthrough by Takahashi and Yamanaka not only provided a valuable cell source for regenerative medicine, but also opened up a new era of further investigation into human developmental biology and stem cell biology. Transcriptomics have characterized global gene expressions at different stages of reprogramming into hiPSCs [7], and this has demonstrated the high degree of similarity between hiPSCs and hESCs. Other high-throughput assays using next generation sequencing have identified the epigenetic signature of hiPSCs, such as DNA methylation and histone modifications. These assays have also addressed multiple features of hiPSCs [9]. An in-depth understanding of cellular pluripotency is necessary for utilizing PSCs in clinical applications.

## 2. STAT3 in Maintenance of Pluripotency

To maintain naïve pluripotency in culture, several different protocols have been established based on the understanding of the molecular network of transcriptional regulation. The leukemia inhibitory factor (LIF) is the first growth factor necessary for the maintenance of mESCs. LIF is a member of the interleukin (IL)-6-cytokine family and binds to the LIF receptor (LIFR), hetero-dimerizing with the signal transducer glycoprotein 130 (gp130). After ligand binding, the LIFR/gp130 complex enhances the kinase activity of JAK, resulting in the subsequent phosphorylation of STAT3. The phosphorylated STAT3 forms homodimers, translocates to the nucleus, and then activates the transcription of the target genes. In embryonic development, the STAT3-knockout mice exhibit embryonic lethality by day 7 post-coitum [10], suggesting that STAT3 activation is crucial in early development. This may underlie a mechanism that mESCs can maintain their undifferentiated state in the presence of STAT3 activation, even without LIF supplementation [11,12]. The link between the LIFR/JAK/STAT3 signaling pathway and the core circuitry of pluripotency-associated transcription factors composed of OCT4, SOX2, KLF4, and NANOG has also been thoroughly analyzed in a previous paper [13]. In mESCs, the JAK/STAT3 pathway regulates KLF4 expression, followed by SOX2 transcription, whereas NANOG is upregulated by TBX3, induced by the phosphatidylinositol-3 kinase (PI3K)/AKT pathway. Similar to the STAT3 overexpression maintaining naïve pluripotency in mESCs, the exogenous expression of KLF4 is sufficient to keep pluripotency. Moreover, the inhibition of the mitogen-activated protein kinase (MAPK), which is another signaling molecule activated by LIFR/gp130, also upregulates TBX3 and NANOG. This is consistent with a report that the MAPK pathway is activated by the fibroblast growth factor (FGF) signal, promoting the ESCs to exit from the pluripotent state [14]. The inhibition of glycogen synthase kinase 3β (GSK3β, a Wnt pathway regulator) also contributes to cell propagation and limiting the differentiation in cultured mESCs [14]. In this regard, a basic formula has been widely used for the maintenance of pluripotency in naïve mESCs. A combination of two kinase inhibitors against GSK3β and MAPK kinase (an upstream molecule of ERK signaling) together with LIF (named as 2i/LIF) is contained in the culture medium [14,15,16,17,18]. Therefore, the regulation of both JAK/STAT and Wnt/GSK3β signaling through LIF-dependent and –independent pathways are crucial to maintain the pluripotent state in mESCs.

Compared to mESCs, hESCs require different growth factors for their maintenance, and they exhibit the distinctive gene expression profile for primed pluripotency. The hESCs need FGF2 and transforming growth factor-β (TGF-β)/activin/nodal signaling to sustain self-renewal and pluripotency [19]. Achieving the conversion from a primed to naïve state in hESCs has been challenging, but is important, as the naïve pluripotency is more immature and can be more widely applied for disease modeling, drug screening, and regenerative medicine. A previous study revealed that the gene introduction of OCT4, KLF4, and KLF2, or the agonist-induced activation of KLF4 and KLF2 in combination with 2i/LIF achieved the entry of hESCs toward the naïve-like state [18,20]. Another study demonstrated that cell culture using 3i (MAPK kinase, GSK3β, and bone morphogenic protein 4 (BMP4) inhibitors) also promotes the responsiveness of hESCs to LIF to acquire naïve-like pluripotency through the upregulation of gp130 [21]. More recently, Chen et al. reported that the transient expression of STAT3 contributes to the induction of hESCs toward naïve-like pluripotency that can be maintained in 2i/LIF culture [22]. They showed that temporal STAT3 activation by a tamoxifen-inducible system in combination with LIF stimulation in hESCs sustained their self-renewal capacity and also resulted in genetic and epigenetic characteristics similar to those in mouse naïve ESCs [22]. Therefore, the activation of LIF/STAT3 signaling is critical for hESCs to acquire naïve-like pluripotency independently of FGF2 and TGF-β/activin/nodal signaling.

## 3. STAT3 in Acquisition of Pluripotency

As LIF/STAT3 signaling regulates the maintenance of the self-renewal and pluripotent properties in ESCs, the role of STAT3 in somatic cell reprogramming has also been documented in several studies focusing on the regulation of LIFR/gp130 signaling. The LIF/STAT3 axis has been demonstrated to complete the reprogramming of mEpiSCs, neural stem cells, and partially reprogrammed cells [23]. STAT3 is known to directly bind to the promoters of the genes constituting the core pluripotent circuitry, such as OCT4 and NANOG [13,24]. Yang et al. engineered a hybrid granulocyte colony-stimulating factor (G-CSF) receptor (GCSFR)/gp130 Y118F receptor to specifically activate endogenous STAT3 signaling in vitro. G-CSF stimulation to the cells expressing this engineered receptor has the capacity to phosphorylate endogenous STAT3. By utilizing this system, they demonstrated that STAT3 activation is required for the conversion from mEpiSCs to naïve iPSCs [23,25]. The involvement of LIF/STAT3 signaling in the epigenetic regulation of reprogramming to pluripotency was also proposed by a previous study [26]. Tang et al. performed experiments using mouse embryonic fibroblasts and showed that a constitutively active form of STAT3 promoted the reprogramming induced by the transgene of OCT4, SOX2, and KLF4 (OSK), or OCT4, SOX2, KLF4, and c-MYC (OSKM). They also found that the inhibition of JAK/STAT3 signaling blocked the demethylation of the OCT4 and NANOG regulatory elements accompanied by a significant increase in DNA methyltransferase 1 (DNMT1) and histone deacetylases (HDACs) expressions. In contrast, the treatment of the DNMT or HDAC inhibitor rescued the reprogramming efficiency repressed by the inactivation of JAK/STAT3 signaling [26].

**Figure 1:**
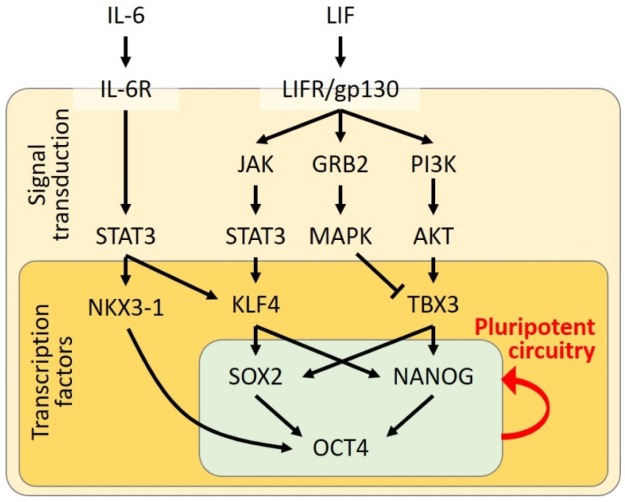
Diagram of the signal transduction activating the pluripotent circuitry of the core transcription factors. The black arrows indicate activation of the downstream molecules.

Furthermore, a recent discovery by Mai et al. revealed that NKX3-1, a homeobox transcription factor, is transiently expressed in the early phase of reprogramming and that it can be used as a substitute for OCT4 for mouse and human iPSC induction [27]. They found that the dynamic expression pattern of NKX3-1 mirrored that of the IL-6 receptor (IL-6R) during heterokaryon reprogramming; they also observed that IL-6R signaling is essential to iPSC reprogramming. Mai et al. further confirmed that the NKX3-1-dependent mechanism of iPSC reprogramming required STAT3 signaling as a direct downstream target of IL-6R. NKX3-1, thus, acts as a downstream target of the IL-6/STAT3 axis during OSKM reprogramming. Figure 1 is an illustration that summarizes the molecular regulatory network of LIF signaling involved in the acquisition and maintenance of the pluripotent circuitry.

## 4. STAT3 in Cardiomyocyte Differentiation from Pluripotent Stem Cells

PSCs have long been a focus in practical regenerative medicine, particularly for any organ comprising of terminally differentiated cells, such as cardiomyocytes or neurons, in which the proliferation capacity is considerably limited. Initially, the differentiation protocol of ESCs resulted in the formation of the embryoid body (EB), which contains variably differentiated cell types of all three germ layers, including beating cardiomyocytes. Although the beating cell aggregate exhibits cardiomyocyte features, including cardiac marker molecules, intracellular calcium oscillation, and contractile property, the incidence of the beating cell population was significantly low among the whole cell population [28]. To efficiently obtain cardiomyocytes ex vivo, researchers to date have made huge efforts, using chemical compounds, microRNA, and cytokines that facilitate cardiomyocyte differentiation from PSCs [29,30,31,32,33,34,35]. For example, the efficiency of cardiomyocyte differentiation can be improved by adding BMP4, WNT3A, and the subsequent supplementation of a WNT signaling inhibitor [36,37,38]. G-CSF and L-ascorbic acid are also known factors to improve cardiac differentiation efficiency by facilitating cardiac progenitor cell propagation [39,40], whereas insulin-like growth factor 1 (IGF1) and IGF2 are reported to stimulate the proliferation capacity in hPSC-derived cardiomyocytes [41].

In terms of the role of STAT3 in cardiac development, a previous comprehensive study demonstrated that in the embryonic heart, there are high expression levels of G-CSF and GCSFR, an upstream signaling molecule of STAT3 at the midgestational stage and onward [40]. G-CSF is a hematopoietic cytokine that stimulates neutrophil colony growth [42,43], and positively regulates stem cell mobilization [44,45]. According to the study by Shimoji et al., intrauterine treatment by G-CSF causes cardiac hyperplasia due to high cardiomyocyte proliferation, whereas deficiency of the GCSFR during the late stages of embryogenesis causes embryonic death because of myocardial thinning. They also found high expression levels of GCSFR in primate ESC- and hiPSC-derived cardiomyocytes, and showed the enhancement of myocardial cell proliferation by G-CSF treatment [40]. These findings suggest the crucial role of G-CSF/JAK/STAT3 signal activation in cardiomyocyte proliferation in the developing heart and PSC-derived cardiomyocytes.

## 5. STAT3 in Heart Disease

The therapeutic roles of STAT3 in heart disease have been reported in previous studies using several models of heart failure, including gene-altered mice and cultured cardiomyocytes. The majority of these studies demonstrate that STAT3 is cardioprotective in pathological conditions through modulating cell survival/death, cell proliferation, neovascularization, and energy metabolism. This beneficial effect of STAT3 may lead to the development of a novel therapeutic strategy for heart disease.

### 5.1. STAT3 in Myocardial Infarction

Ischemic heart diseases, such as myocardial infarction, are a major cause of cardiac death in humans. The occlusion of coronary arteries by atheroma and thrombus disrupts the blood supply to the distal myocardium, resulting in cell death. In addition to the importance of STAT3 in myocardial differentiation from hPSCs, STAT3 plays a pivotal role in cardiomyocyte survival in adult hearts. As an upstream factor of STAT3, G-CSF has been known to promote myocardial regeneration through inducing the mobilization of bone marrow stem cells (or bone marrow stromal cells: BMSCs), a type of multipotent mesenchymal stem cell, to the injured tissue [46,47,48,49,50,51,52]. Although mobilized BMSCs had been thought to differentiate to cardiomyocytes in the infarcted heart, a series of studies published in later years suggested that BMSCs contribute to cardiac regeneration by promoting residual cardiomyocyte survival, possibly owing to the paracrine effect [53,54,55]. In addition, a recent study by Cai et al. supported the evidence that the myocardial differentiation from BMSCs occurs via miRNA-involved modulation [56]. They reported that miR-124 inhibition activates STAT3 and enhances differentiation efficiency from BMSCs, whereas miR-124 overexpression reduced the efficiency [56]. Thus, although there is still controversy regarding the underlying mechanism of BMSC-based myocardial repair, BMSCs have advantages, including relatively high availability and proliferation capacity, as well as multipotent property and the secretion of growth factors and cytokines [57]. BMSCs, in comparison with PSCs, are also expected as another optimal cell source in regenerative medicine. Uncovering the mechanisms involving miRNAs would provide novel insights into cardiac differentiation from BMSCs, thereby facilitating the clinical application of BMSC-based regeneration therapy.

Harada et al. reported that GCSFR signaling is therapeutic in cardiomyocytes and non-cardiomyocytes in an injured heart [58]. The underlying mechanism was that G-CSF administration prevents post-infarction ventricular remodeling through the activation of the JAK/STAT pathway. The beneficial effect of G-CSF administration occurs in the early phase after infarction. The G-CSF/JAK/STAT-mediated improvement of cardiac function is attributed to the upregulation of Bcl-2 and Bcl-xL (anti-apoptotic molecules), leading to the inhibition of the apoptosis of the ischemic myocardium and the survival of endothelial cells necessary for neovascularization. A previous study using AG490, a JAK/STAT pathway inhibitor, also supports the above findings [59]. Moreover, loss-of-function experiments using transgenic mice overexpressing a dominant-negative mutant of STAT3 in cardiomyocytes showed that STAT3 is a key molecule for the G-CSF-mediated cardiomyocyte survival and the prevention of ventricular remodeling [58]. In addition, the cardiac-specific knockout of STAT3 exacerbated ventricular remodeling during the subacute phase of myocardial infarction in mice [60]. According to a previous study, cardioprotective roles of STAT3 are involved in the suppression of miR-199a-5p transcription, because miR-199a-5p disrupts protein turnover in cardiomyocytes and elevates oxidative stress in cardiac endothelial cells [61]. In terms of the STAT3 effects on endothelial cell survival promoting neovascularization, this is also supported by the evidence that the JAK/STAT pathway induces angiogenic factors, which has been investigated in cardiac and cancer research fields [62,63,64,65].

### 5.2. STAT3 in Ischemic/Reperfusion Injury

In acute coronary syndrome leading to life-threatening myocardial infarction, recanalization with catheter intervention causes ischemia/reperfusion injury, owing to excessive oxidative stress. Similar to the role of STAT3 in myocardial infarction, STAT3 is protective against ischemia/reperfusion injury by decreasing oxidative stress, apoptosis, and mitochondrial dysfunction, and by increasing angiogenesis [66,67]. This is supported by a study using STAT3-deficient mice exhibiting severe myocardial damage following ischemia/reperfusion [68,69]. Oxidative stress is elevated by the increased generation of reactive oxygen species and/or decreased antioxidant production. STAT3 is known to increase the level of antioxidant metallothioneins and manganese superoxide dismutase (MnSOD), whereas STAT3 decreases the production of reactive oxygen species by the modulation of complexes I and III of the electron transport chain [66,67,70]. 

### 5.3. STAT3 in Doxorubicin-Induced Cardiomyopathy

Doxorubicin is widely used in cancer chemotherapy and commonly known to have cardiotoxicity if its accumulated dose exceeds 550 mg/m^2^ [71,72]. The underlying mechanisms of doxorubicin-mediated cardiotoxicity have been demonstrated as follows: (1) excessive oxidative stress, (2) impaired mitochondrial iron transport and mitochondrial dysfunction, and (3) topoisomerase II inhibition and DNA damage [71,73,74]. All of these result in cell death, leading to heart failure. In particular, many studies supported the conclusion that oxidative stress corresponds to the pathophysiology of doxorubicin-induced cardiomyopathy. As mentioned, STAT3 plays a role in reducing oxidative stress in the physiological and pathological conditions of the heart. There are several studies that found myocardial protection by STAT3 against doxorubicin-induced cardiomyopathy. The cardiac-specific overexpression of STAT3 resulted in a significant increase in the survival rate following doxorubicin administration and maintained the expression of hypertrophy-responsive genes, such as the atrial natriuretic factor, β-myosin heavy chain, and cardiotrophin-1 genes. As STAT3 gene expression was downregulated in the heart of doxorubicin-treated mice, STAT3 potentially protected the myocardial tissue from doxorubicin toxicity [75]. A couple of molecular mechanisms of STAT3-mediated protection against doxorubicin have been proposed so far. Rong et al. reported that JAK2/STAT3 activation increases the expression of metallothionein 1 and 2 anti-oxidative genes in response to doxorubicin stimulation, thereby decreasing oxidative stress [76]. Another report by Wu et al. demonstrated that S-propargyl-cysteine, an endogenous hydrogen sulfide initiator, is cardioprotective against doxorubicin-induced toxicity through STAT3 activation by gp130-dependent signaling [77].

### 5.4. STAT3 in Cardiac Fibrosis and Hypertrophy

In addition to the protective effect, STAT3 is involved in cardiac fibrosis and hypertrophy. In the physiological condition, in vitro experiments revealed that STAT3 is activated in cardiac fibroblasts by IL-6 involved in normal collagen synthesis to maintain tissue homeostasis [78]. The upregulation of IL-6 in cardiomyocytes was observed in a hypertension animal model through renal artery ligation, showing significantly increased cardiac fibrosis [79]. Moreover, the treatment of angiotensin II—a pathological stimulus—in cultured cardiac fibroblasts induces STAT3 activation via Rac1 indirectly (paracrine effect), leading to increased fibrosis [80]. Another previous study also reported that the activation of the gp130/STAT3 pathway by LIF treatment induces cardiomyocyte hypertrophy, and this effect was blunted by SOCS3, a suppressor of cytokine signaling [81]. These studies suggest the link between the cytokine-mediated inflammation response in fibroblasts and STAT3-involved hypertrophy in cardiomyocytes. Interestingly, a recent study showed that STAT3-dependent hypertrophy is at least in part regulated by the inhibition of cellular autophagy [82]. In H9c2 cells (mouse atrial myocyte line), angiotensin II induced hypertrophy via JAK/STAT3 signal activation, and this hypertrophic response was abolished by the knockdown or pharmacological inhibition of STAT3 through increased autophagy-related proteins and decreased phosphorylated AMP-activated protein kinase α (AMPKα) and mammalian target of rapamycin (mTOR). These findings suggest that STAT3 contributes to a balance between autophagy and hypertrophy in response to angiotensin II stimulation [82]. Furthermore, STAT3 has been shown to regulate various mitochondrial functions involved in energy metabolism in the heart. The healthy heart relies on fatty acids rather than glucose for fuel. Altara et al. demonstrated that chronic hypertension induced by angiotensin II infusion tends to switch the energy metabolism toward glycolysis from fatty acid oxidation in cardiac-specific STAT3 knockout mice [83], suggesting the role of STAT3 in metabolism regulation.

Several studies reported that the deficiency of gp130 in pressure-overloaded hypertrophy model mice exacerbated heart failure through the inactivation of STAT3. These mice displayed the early onset of dilated cardiomyopathy and a low survival rate without an increase in hypertrophy or fibrosis [84,85,86]. Another study using cardiac-specific STAT3 knockout mice demonstrated that myocardial infarction increased myocardial fibrosis associated with the upregulation of fibrosis-related genes and enhanced cardiomyocyte hypertrophy [60]. Although either the overactivation or inhibition of STAT3 appears to exacerbate cardiac pathophysiology, the fibrotic and remodeling responses would vary among types of heart failure. Thus, for proper heart failure treatment, STAT3 activation should be fine-tuned at the physiological level.

## 6. STAT3 Activation for Myocardial Regeneration

Despite the cardiomyocyte differentiation from PSCs to replace damaged tissue, STAT3 activation promotes myocardial regeneration in residual living tissue. The protective roles of the JAK/STAT pathway, including proliferation, survival, and cell competition, are evolutionally conserved among vertebrates and Drosophila. Several studies have demonstrated the JAK/STAT-mediated regeneration of various tissues in Drosophila, such as the intestine, wing disc, and testis [87,88]. Similarly, it is known that the myocardium in amphibians and fish can be completely regenerated by the proliferation of mature cardiomyocytes after injury, even in adult creatures [89,90]. In this context, it was also reported that the regeneration of zebrafish tissues, including cardiomyocytes, is promoted by JAK/STAT signal activation [91,92,93,94]. Fang et al. investigated the underlying mechanism of injury-induced heart regeneration in zebrafish [91]. Using translating ribosome affinity purification (TRAP), a special technology for the profiling of actively translated mRNAs in a specific cell type, they discovered that protein expressions associated with the Jak1/Stat3 axis are dynamically induced following tissue injury. Using transgenic zebrafish, in which cardiac Stat3 is inhibited by dominant-negative Stat3, they also confirmed that Jak1/Stat3 stimulates cardiomyocyte proliferation and regeneration. The activation of the Jak1/Stat3 pathway by injury induces the secretion of Rln3a and interleukin 11α (Il11α). Rln3a is an orthologue of mammalian relaxin3, a peptide hormone acting against myocardial injury, oxidative stress, fibrosis, and inflammation in the cardiovascular system, and known to be required for cardiomyocyte proliferation [95]. Il11α is a ligand of the Jak1/Stat3 pathway that is produced from the endocardium. Based on these findings, they proposed a dynamic flow of the mechanism as follows: 1) Jak1/Stat3 downstream mediators are activated at the damaged myocardium; 2) this promotes cytokine production in the endocardium and inflammatory cells in the whole heart; 3) these cytokines are then localized and activated at the injury site, where they stimulate Rln3a production [91]. The secreted Il11α may also be cardioprotective, as reported in mice with myocardial infarction [96].

As observed in zebrafish, myocardial regeneration in response to injury has also been reported in neonatal mice [97]. The regeneration capacity of the myocardial tissue declines during the first week after birth, and afterward, cardiomyocytes exit the cell cycle. Then, cardiomyocytes begin to grow by hypertrophy to become mature [98]. A terminally differentiated adult mammalian heart displays a limited capacity for self-renewal when damaged [99,100,101]. In terms of mechanisms underlying myocardial regeneration in neonatal mice, a recent comprehensive study examined how the transcriptional characteristics of differentiated myocytes reverts to the immature form during regeneration in response to injury [102]. O’Meara et al. analyzed dynamic transcriptional changes during myocardial regeneration after apex resection in mouse neonates. They revealed a transcriptional reversion of differentiation processes, such as the reactivation of cell cycle genes and developmental programs. In vitro and in vivo cardiomyocyte differentiation and the explant myocyte model also displayed similar dynamic profile changes. The study further identified that interleukin 13 (IL-13) is a potential upstream regulator for the transcriptional reversion during regeneration that induced STAT3/periostin and STAT6 signaling, as well as the cell cycle entry of cardiomyocytes [102]. Periostin is a component of the extracellular matrix and is induced after myocardial injury [103,104,105]. As periostin delivery promotes myocyte proliferation and reduces injury size and fibrosis in myocardial infarction model rats [106], IL-13 presumably facilitates STAT3 and periostin induction for heart regeneration. Altogether, the cardiac-specific activation of the STAT3 pathway by ligand stimulation would be a favorable strategy for cardiac regeneration therapy.

In the clinical setting, there are several therapeutic strategies for cardiac repair, including pharmacological, gene-based, and cell-based therapy. G-CSF administration, which could trigger STAT3-mediated myocardial regeneration as mentioned, has been performed to improve cardiac function in patients with angina and/or acute myocardial infarction [107]. The gene delivery of a vascular endothelial growth factor or hepatocyte growth factor has also been evaluated in several clinical trials, showing only modest-to-partial effects for the treatment of ischemic heart disease [108,109,110,111]. Beside cytokine- or growth factor-based approaches, cell-based cardiac regeneration therapy has been investigated in many clinical trials for the last 20 years. BMSCs were commonly used for cell transplantation via intracoronary catheter intervention or direct myocardial injection, and some studies showed the effectiveness in failing hearts [112]. In reality, however, their outcome was controversial due to different study conditions, including the types and numbers of cells to be transplanted, administration methods, lack of control, and variations of disease type, disease stage, and end-point, which were not standardized [112]. Even several well-designed clinical studies did not show a significant improvement of the cardiac function [113,114]. Therefore, further clinical trials must be standardized to reliably evaluate the feasibility, efficiency, and safety of cell-based cardiac regeneration therapy. Recently, the first clinical trial to treat severe heart failure using a tissue sheet of iPSC-derived cardiomyocytes was approved and started [115]. The clinical outcome of this new type of cell therapy may contribute to deciding the future of cardiac regenerative medicine.

Considering the limited effects of stem cell-based therapy on clinical outcome, another therapeutic strategy, direct cardiac reprogramming, was developed to apply for myocardial regeneration. Ieda et al. first reported the direct conversion from fibroblasts to cardiomyocytes in vitro by the gene introduction of Gata4, Mef2c, and Tbx5, which are cardiac-specific transcription factors [116]. Later, Qian et al. and Inagawa et al. demonstrated that the intramyocardial injection of Gata4-, Mef2c-, and Tbx5-expressing retroviral vectors succeeded to reprogram residual non-myocytes to cardiomyocytes and improved cardiac function after myocardial infarction in mice [117,118]. Although further studies have been conducted to enhance the reprogramming efficiency using additional transcription factors (e.g., Hand2), a combination of miRNAs (e.g., miR-1, miR-133, and miR-408), or other type of viral vectors (e.g., Sendai virus) [119,120,121], the efficacy of direct reprogramming to cardiomyocytes is still being improved. Therefore, uncovering the mechanisms underlying direct reprogramming as well as cytokine-, growth factor-, and cell-based cardiac regeneration therapy will shed light on further development of these therapeutic options and the current knowledge of the molecules involved in cardiac regeneration, such as STAT3, would help to accelerate the advancement. Table 1 is a summary of STAT3-mediated diverse functions in early embryos, stem cells, and developing and diseased hearts.

## 7. STAT3 Activation through Artificial Receptors for Myocardial Differentiation

Stem cell-based myocardial regeneration therapy for heart failure remains challenging despite the extensive efforts made over the last decade. Most protocols to induce cardiomyocytes from PSCs require growth factors or chemical compounds, some of which are expensive [31,32,34,123,124]. This financial hurdle limits clinical application, since a huge number of cardiomyocytes are needed for cell therapy. To overcome this problem, recent bioengineering technologies have enabled us to develop artificial receptors that can respond to an inexpensive surrogate ligand. For STAT3 activation, Kawahara and colleagues have developed several artificial cytokine receptors, named signalobodies, to control cellular functions. The scFv-c-Fms (S-Fms) signalobody activates downstream signaling molecules, including MEK, ERK, AKT, and STAT3 [125,126,127]. A heterodimeric signalobody of V_H_/EpoR and V_L_/gp130 could also induce the activation of JAK/STAT signaling [128].

**Figure 2:**
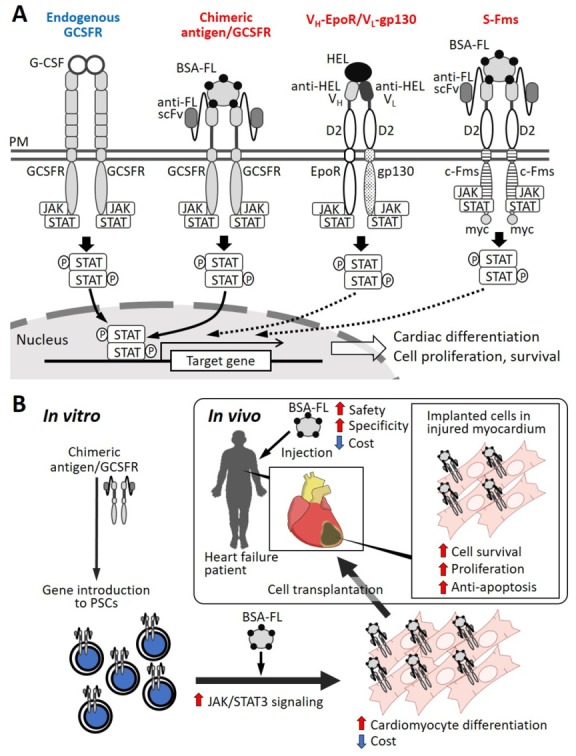
(**A**). Schematic illustrations of artificial receptors activating STAT signaling in response to a surrogate ligand (BSA-FL or HEL). (**B**). A strategy of cardiac regeneration therapy based on transplantation of cardiomyocyte-derived from chimeric antigen/GCSFR-expressing PSCs. BSA-FL: fluorescein-conjugated bovine serum albumin; D2; extracellular D2 domain of erythropoietin receptor; EpoR: erythropoietin receptor; G-CSF: granulocyte colony-stimulating factor; GCSFR: G-CSF receptor; HEL: hen egg lysozyme; JAK: Janus kinase; PM: plasma membrane; scFv: single chain Fv; STAT: signal transducer and activator of transcription; V_H_: variable region of heavy chain; V_L_: variable region of light chain.

Using the above technologies, we recently reported a novel strategy to improve the differentiation efficiency from miPSCs to cardiomyocytes by the economical activation of G-CSF/STAT3 signaling in response to a surrogate ligand [122]. As mentioned in the previous section, G-CSF is a well-known hematopoietic cytokine that regulates stem cell mobilization and anti-apoptosis via JAK/STAT signal activation [42,43,129,130]. It was also reported that G-CSF stimulation promotes cardiomyocyte differentiation from ESCs and iPSCs [40]. Although recombinant G-CSF is clinically used for chemo-associated neutropenia treatment in cancer patients, G-CSF is expensive for repetitive usages. Thus, we constructed a chimeric antigen/GCSFR responsive to cognate artificial antigens, fluorescein-conjugated bovine serum albumin (BSA-FL) [122]. BSA-FL is inexpensive, chemically stable, and physiologically inactive compared to recombinant G-CSF, and it is expected to bind to this artificial receptor, specifically activating the downstream signaling pathways in the targeted cells. Regarding the molecular structure of the chimeric antigen/GCSFR, an anti-FL single-chain Fv (scFv) was fused to the transmembrane/intracellular domains of the native GCSFR that could trigger endogenous JAK/STAT signal activation following the ligand binding. We then generated a stable miPSC line introduced with chimeric antigen/GCSFRs. The administration of BSA-FL as an inexpensive surrogate ligand (about 20 times less expensive than recombinant G-CSF) in this cell line successfully and dose-dependently triggered STAT3 phosphorylation, and, in turn, improved the efficiency of cardiomyocyte differentiation demonstrated by an increased incidence of beating EB formation. The beating EBs also exhibited the upregulation of cardiac transcription factors and structural molecules, such as TBX5, GATA4, α-actin, and α-myosin heavy chain, whereas other mesodermal marker gene expressions seemed not to be affected by BSA-FL treatment. There was a downregulation of ecto- and endo-dermal marker genes by BSA-FL. In addition, JAK Inhibitor I perturbed BSA-FL-mediated increases in the STAT3 phosphorylation and myocardial differentiation efficiency. These findings suggest that the artificial GCSFR-mediated signal transduction in response to a surrogate ligand activates the JAK/STAT pathway, contributing to differentiation preferential to cardiomyocytes rather than to other cell types [122]. Therefore, the artificial GCSFR we constructed would be a favorable and economical tool to enhance the cardiomyocyte creation from PSCs (Table 1). Furthermore, applying cardiomyocytes derived from artificial GCSFR-expressing PSCs, together with the systemic administration of surrogate ligands instead of recombinant G-CSF, could be beneficial after cell implantation, considering the effects of the JAK/STAT pathway activation on myocardial regeneration, as discussed above [58,91,102]. As the phosphorylation levels of STAT3 depend on the ligand dose, stimulating the artificial GCSFR by a proper dose of BSA-FL could finely tune the activity of the JAK/STAT pathway. Further investigations will optimize the temporal activation of STAT3, using engineered chimeric receptor-expressing cells for effective myocardial regeneration with minimal fibrosis. In Figure 2A, we summarize the structure and signal transduction of several types of artificial chimeric antigen receptors that can trigger JAK/STAT signal activation in response to surrogate ligands; we also show the strategy of cardiac regeneration therapy based on the transplantation of cardiomyocytes derived from the artificial GCSFR-expressing PSCs (Figure 2B).

## 8. Conclusions and Perspectives

Cardiac regenerative medicine is currently a high hope because heart disease is the leading cause of death in the developed countries. Although serious research has been conducted, obtaining the sufficient number and quality of cardiomyocytes derived from PSCs still presents challenges. Therefore, it is important to identify key molecules and signal pathways, such as JAK/STAT3 signaling for the induction, maintenance, and differentiation of PSCs, while a numerous number of cytokines and secretary factors, such as G-CSF, have also been screened to underpin their utility for cardiac regeneration therapy. Accumulating evidence revealed that: (1) STAT3 maintains pluripotency in naïve mouse and human PSCs as a downstream regulator of LIF; (2) STAT3 promotes proliferation in the developing PSC-derived cardiomyocytes; (3) in adult hearts, STAT3 plays a role in cardioprotection through G-CSF and gp130 signal activation in various cardiovascular diseases; (4) STAT3 is involved in myocardial regeneration mediated by the dedifferentiation and proliferation of cardiomyocytes, observed in the myocardium of neonatal rodents, as well as amphibians and fish. Cardiac regeneration therapy relies on a couple of issues to be achieved: stem cell differentiation, the re-entry and enhancement of cell cycle progression, the prevention of cell death, and improving the function of residual myocytes. Although STAT3 is involved in most of them, its overactivation is rather harmful to the heart because it may lead to cardiac fibrosis. Therefore, it is important to develop the strategies to regulate STAT3 activity. In this context, the artificial activation of the JAK/STAT3 pathway at proper levels by chimeric antigen/GCSFR, using an inexpensive surrogate ligand, would possibly be a powerful tool to establish an economical and innovative cardiac regeneration therapy.

## Figures and Tables

**Table 1:** STAT3-mediated biological functions in early embryos, stem cells, and developing and diseased hearts.

Ligand/Stimulus	Receptor/Effector	Cell/Tissue Type	Functions	Mechanisms	References
LIF	LIFR/gp130	mouse early embryo	early development	possible role in visceral endoderm	[10]
LIF	LIFR/gp130	mESC	pluripotency maintenance	induction of KLF4 expression subsequently activating SOX2 transcription	[11,12,13]
LIF	LIFR/gp130	hESC	naïve pluripotency acquisition	NA	[22]
G-CSF	GCSFR/gp130 Y118F chimeric receptor	mEpiSC	naïve pluripotency acquisition	NA	[23,25]
LIF	LIFR/gp130	MEF	reprogramming to miPSCs	demethylation and deacetylation of OCT4 and Nanog	[26]
IL-6	IL6R	MEF, human fibroblast	reprogramming to miPSCs and hiPSCs	activation of activates endogenous OCT4 by NKX3-1	[27]
G-CSF	GCSFR	mouse embryonic heart	cardiac development	cardiomyocyte proliferation	[40]
G-CSF	GCSFR	BMSC (mouse, rabbit, human)	cardioprotective against MI	mobilization to injured myocardium	[46,47,48,49,50,51,52]
G-CSF	GCSFR	BMSC (mouse, non-human primate)	cardioprotective against MI	cardiomyocyte survival as paracrine effect	[53,54,55]
NA	miR-124	BMSC (rat)	pathological factor	inhibition of cardiomyocyte differentiation	[56]
G-CSF	GCSFR	mouse and rat cardiomyocyte	cardioprotective against MI	anti-apoptosis, prevention of ventricular remodeling	[58,59,60]
G-CSF	GCSFR	mouse and rat endothelial cell	cardioprotective against MI	cell survival, neovascularization	[58,59,60]
NA	miR-199-5p	mouse and rat cardiomyocyte	pathological factor	disruption of protein turnover	[61]
NA	miR-199-5p	mouse and rat endothelial cell	pathological factor	oxidative stress elevation	[61]
NA	NA	adult mouse heart	cardioprotective against I/R injury	decreasing of oxidative stress, apoptosis and mitochondrial dysfunction; increasing angiogenesis	[67,68,69]
NA	NA	adult mouse heart	cardioprotective against I/R injury	increase in antioxidants (metallothioneins, MnSOD): decrease in ROS production via complexes I and III activation	[66,67,70]
NA	NA	adult mouse heart	cardioprotective against doxorubicin-induced cardiomyopathy	cell survival, increase in antioxidants (metallothionein 1 and 2)	[76]
NA	gp130	adult mouse heart	cardioprotective against doxorubicin-induced cardiomyopathy	in response to S-propargyl-cysteine (hydrogen sulfide initiator)	[77]
IL-6	NA	rat cardiac fibroblast	physiological and pathological fibrosis	collagen synthesis	[78,79]
Angiotensin II /Rac1	NA	rat cardiac fibroblast	fibrosis	collagen synthesis	[80]
LIF	LIFR/gp130	mouse and rat cardiomyocyte	hypertrophy	cytokine-mediated hypertrophy and anti-apoptosis	[81]
Angiotensin II	NA	H9c2 cell line	anti-hypertrophy	inhibition of autophagy-related proteins; activation of AMPKα and mTOR	[82]
NA	NA	adult mouse heart	cardioprotection against hypertension	inhibition to shift energy metabolism from fatty acid oxidation to glycolysis	[83]
NA	gp130	adult mouse heart	cardioprotection against early onset of dilated cardiomyopathy induced by pressure overload	anti-apoptosis	[84,85,86]
Il11α	NA	zebrafish heart	myocardial regeneration after injury	cardiomyocyte proliferation through cytokine production in endocardium and inflammatory cells	[91,92,93,94]
IL-11	NA	adult mouse heart	cardioprotective against MI	Prevention of apoptosis, fibrosis and ventricular remodeling; neovascularization	[96]
IL-13	NA	neonatal mouse heart	myocardial regeneration after injury	reversion of transcription profiles for cardiomyocyte development and maturation	[102]
Surrogate ligand (BSA-FL)	chimericantigen/GCSFR	miPSC	cardiomyocyte differentiation	NA	[122]

AMPKα, AMP-activated protein kinase alpha; BMSC, bone marrow stem cell (bone barrow stromal cell); BSA-FL, fluorescein-conjugated bovine serum albumin; G-CSF, granulocyte colony-stimulating factor; GCSFR, G-CSF receptor; gp130, glycoprotein 130; hESC, human embryonic stem cell; hiPSC, human induced pluripotent stem cell; IL-6, interleukin 6; IL-6R, IL-6 receptor; IL-11, interleukin 11; Il11α, interleukin 11α; IL-13, interleukin 13; I/R, ischemia/reperfusion; LIF, leukemia inhibitory factor; LIFR, LIF receptor; mEpiSC; mouse epiblast stem cell; mESC, mouse embryonic stem cell; MEF, mouse embryonic fibroblast; MI, myocardial infarction; miPSC, mouse induced pluripotent stem cell; miR, micro-RNA; MnSOD, manganese superoxide dismutase; mTOR, mammalian target of rapamycin; ROS, reactive oxygen species; scFV, single chain FV against fluorescein; NA, not applicable.

## Abbreviations

AMPKα	AMP-activated protein kinase α
BMSC	bone marrow stem cell or bone marrow stromal cell
BSA-FL	fluorescein-conjugated bovine serum albumin
DNMT	DNA methyltransferase
EB	embryoid body
EpiSC	epiblast stem cell
ESC	embryonic stem cell
FGF	fibroblast growth factor
FL	Fluorescein
G-CSF	granulocyte colony-stimulating factor
GCSFR	granulocyte colony-stimulating factor receptor
gp130	glycoprotein 130
GSK3β	glycogen synthase kinase 3β
HDAC	histone deacetylase
hESC	human embryonic stem cells
hiPSC	human induced pluripotent stem cell
hPSC	human pluripotent stem cell
IGF	insulin-like growth factor
IL-6	interleukin 6
Il11α	interleukin 11α
IL-13	interleukin 13
iPSC	induced pluripotent stem cell
JAK	Janus kinase
LIF	leukemia inhibitory factor
MAPK	mitogen-activated protein kinase
mEpiSC	mouse epiblast stem cell
mESC	mouse embryonic stem cell
miPSC	mouse induced pluripotent stem cell
MnSOD	manganese superoxide dismutase
mTOR	mammalian target of rapamycin
OSK	OCT4, SOX2, and KLF4
OSKM	OCT4, SOX2, KLF4, and c-MYC
PI3K	phosphatidylinositol-3 kinase
PSC	pluripotent stem cell
scFv	single chain variable domain of antibody
STAT	signal transducer and activator of transcription
